# Dietary Phytochemicals in Cancer Therapy—From Mechanistic Insights to Combination Strategies

**DOI:** 10.3390/nu18101524

**Published:** 2026-05-11

**Authors:** Ching-Hsein Chen, Yi-Wen Liu

**Affiliations:** Department of Microbiology, Immunology and Biopharmaceuticals, College of Life Sciences, National Chiayi University, A25-303 Room, Life Sciences Hall, No. 300, Syuefu Road, Chiayi City 600355, Taiwan; ywlss@mail.ncyu.edu.tw

## 1. Introduction

Dietary phytochemicals have attracted increasing attention as promising anticancer agents due to their multi-target biological activities and relatively low toxicity [1,2]. Unlike conventional therapies that often focus on single molecular targets, phytochemicals exert pleiotropic effects by modulating interconnected signaling pathways involved in cancer cell survival, proliferation, metabolism, and the tumor microenvironment [3].

This Special Issue, Anticancer Activities of Dietary Phytochemicals: 2nd Edition, gathers eight contributions, including five original research articles and three review articles, that collectively provide new insights into the mechanisms and therapeutic potential of dietary phytochemicals. A central theme emerging from these studies is the critical role of reactive oxygen species (ROS)-mediated stress responses and mitogen-activated protein kinase (MAPK) signaling pathways in mediating the anticancer effects of dietary phytochemicals. This Editorial provides an integrative overview of the key findings and emerging mechanistic themes highlighted in this Special Issue.

## 2. Overview of This Special Issue

The contributions included in this Special Issue highlight the diverse anticancer activities of phytochemicals across multiple cancer types and experimental models. Several studies emphasize the importance of combination strategies in enhancing the therapeutic efficacy of dietary phytochemicals. For instance, the combination of amygdalin and sulforaphane strongly inhibits renal cancer cell proliferation through coordinated modulation of signaling pathways (contribution 1). Similarly, curcumin significantly increases doxorubicin-induced apoptosis compared with that of doxorubicin alone via JNK-dependent MAPK signaling in osteosarcoma cells (contribution 2). In hematologic malignancies, olive leaf extract potentiates the anticancer effects of cytarabine, suggesting its role as a chemosensitizer (contribution 4).

Cucurbitacin B provides insight into the multi-layered mechanisms involving metabolic reprogramming and immune modulation (contribution 3), and the nanoemulsion-based delivery of grape seed oil highlights the importance of increasing bioavailability and therapeutic efficacy (contribution 5). Review articles focusing on shikonin, Gnetin C, and ginseng-derived compounds further expand the mechanistic and translational perspectives on the use of phytochemicals in cancer therapy (contributions 6–8).

## 3. ROS and MAPK Signaling as Central Mechanistic Themes

The studies included in this Special Issue collectively highlight the central role of ROS-mediated stress responses in phytochemical-induced anticancer activity, which are key mechanisms in cancer therapy [4]. Several contributions demonstrate that increased ROS levels act as an upstream trigger for activating stress-responsive signaling pathways, particularly MAPK cascades, thereby promoting apoptotic responses (contributions 2, 3, and 6). 

In this context, MAPK signaling emerges as a key convergence node linking oxidative stress to downstream cellular responses. The activation of the JNK and p38 pathways has been consistently associated with apoptosis induction. The modulation of ERK signaling exhibits context-dependent roles in regulating cell survival and death (contributions 1 and 2). These findings underscore a shared mechanistic framework across phytochemicals and cancer models.

The cross-talk between MAPK pathways and survival signaling networks further refines our understanding of the cellular responses to cancer treatment. Inhibiting pro-survival pathways, such as the PI3K/Akt pathway, enhances the pro-apoptotic effects of MAPK activation, thereby contributing to the overall therapeutic outcome (contribution 1).

## 4. Combination Strategies as a Core Therapeutic Concept

A key insight from this Special Issue is that phytochemicals exert enhanced anticancer effects when applied in combination strategies than when applied alone, an approach that is a promising direction in cancer therapy [5]. These combinations may involve phytochemical–chemotherapy interactions or phytochemical–phytochemical synergy, both of which contribute to increased therapeutic efficacy.

Phytochemical–chemotherapy combinations improve treatment outcomes by amplifying oxidative stress and modulating stress-responsive signaling pathways. For example, curcumin enhances doxorubicin-induced apoptosis through JNK activation, and olive leaf extract increases the efficacy of cytarabine (contributions 2 and 4). These findings suggest that phytochemicals function as sensitizers that lower the apoptotic threshold and enhance drug responsiveness.

Phytochemical–phytochemical combinations similarly enable the coordinated targeting of complementary signaling pathways. The combination of amygdalin and sulforaphane illustrates how the modulation of two molecular pathways can result in synergistic anticancer effects (contribution 1). Such strategies reduce the likelihood of compensatory signaling and provide broader-spectrum activity against tumor cells.

**Figure 1:**
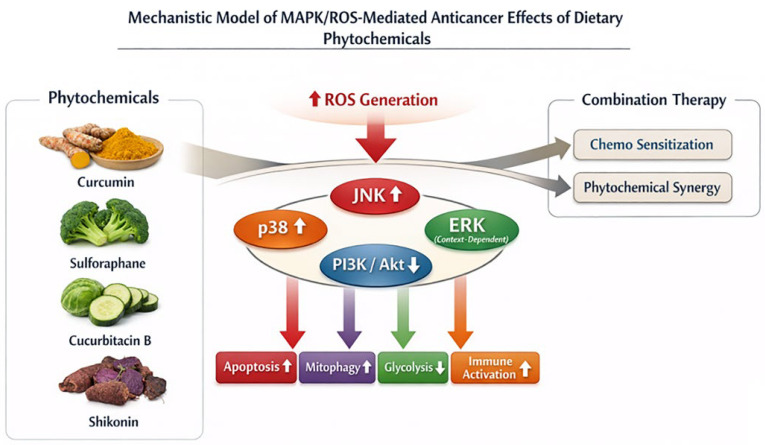
Proposed mechanistic model illustrating anticancer effects of dietary phytochemicals through ROS-mediated MAPK signaling pathways. Phytochemicals induce ROS generation, activating MAPK pathways (JNK, p38, ERK) and suppressing PI3K/Akt signaling. These effects lead to apoptosis, mitophagy, glycolysis, and immune activation, which are further enhanced under combination therapy conditions. This model highlights the integrated and multi-target nature of phytochemical-mediated anticancer mechanisms.

Taken together, these findings support a unified mechanistic model in which combination therapy converges on the ROS-mediated activation of MAPK pathways and the simultaneous suppression of survival signaling, as illustrated in Figure 1, which summarizes the integrated signaling framework underlying these mechanisms. This dual regulation promotes a coordinated shift toward cancer cell death and enhances therapeutic efficacy.

## 5. Tumor Metabolism and Microenvironment

In addition to their direct cytotoxic effects, several contributions highlight the role of phytochemicals in regulating tumor metabolism and the tumor microenvironment. Metabolic reprogramming, particularly enhanced glycolysis, is a hallmark of cancer that supports rapid cell proliferation and survival. The evidence is increasingly indicating that phytochemicals regulate metabolic processes [6].

Cucurbitacin B provides a representative example of how phytochemicals can disrupt metabolic homeostasis by inhibiting glycolysis and inducing mitophagy, thereby impairing tumor energy metabolism (contribution 3). In addition, the modulation of metabolic pathways by cucurbitacin B is closely linked to immune regulation, as alterations in lactate production and redox balance can influence immune cell function and tumor progression.

These findings suggest that phytochemicals act as not only direct anticancer agents but also regulators of the tumor microenvironment, contributing to a more comprehensive therapeutic effect.

## 6. Translational Perspectives

Despite the promising anticancer activities of phytochemicals, the clinical application of phytochemicals remains limited by challenges related to bioavailability and stability. Several contributions highlight the importance of formulation strategies in overcoming these limitations.

Nanoformulation approaches, such as nanoemulsion-based delivery systems, can enhance the bioavailability and therapeutic efficacy of phytochemicals (contribution 5). In addition, the structural optimization of compounds, such as Gnetin C and shikonin, further supports the translational potential of phytochemicals (contributions 6 and 7).

These advances underscore the importance of integrating mechanistic insights with delivery technologies to facilitate the clinical translation of phytochemicals. Despite these advances, challenges related to bioavailability and pharmacokinetics remain major barriers to the clinical application of phytochemicals [7].

## 7. Future Perspectives

The findings presented in this Special Issue highlight several important directions for future research. First, further studies are needed to validate the anticancer efficacy of phytochemicals in clinical settings. Second, a deeper understanding of the molecular mechanisms underlying phytochemical activity will be essential for optimizing their therapeutic application. In particular, the integration of phytochemicals with emerging therapeutic strategies, such as immunotherapy and targeted therapy, represents a promising avenue for enhancing treatment outcomes. Advances in delivery systems, combined with mechanism-based approaches, may further enable the clinical applicability of these compounds. These advances will be critical for translating phytochemical-based strategies into clinically applicable cancer therapies.

## 8. Conclusions

The studies included in this Special Issue collectively demonstrate that dietary phytochemicals are highly promising multi-target anticancer agents capable of modulating complex signaling networks. A central theme emerging from these contributions is the critical role of ROS-mediated stress responses and MAPK signaling pathways in regulating cancer cell fate.

Phytochemicals exert their strongest therapeutic effects when applied in combination strategies that simultaneously enhance pro-apoptotic signaling and suppress survival pathways. In addition, the ability of phytochemicals to modulate tumor metabolism and the tumor microenvironment further expands their functional role in cancer therapy.

These findings together support a conceptual framework in which dietary phytochemicals act as integrative modulatojp65; jors of cancer-related pathways. This integrated approach may ultimately bridge the gap between experimental findings and clinical application, thereby accelerating the translation of dietary phytochemicals into evidence-based cancer therapies. The major findings and mechanisms of the studies included in this Special Issue are summarized in Table 1.

## Figures and Tables

**Table 1:** Summary of studies included in this Special Issue.

Contribution	Compound/Strategy	Cancer Type	Mechanisms	Key Feature
1	Amygdalin + SFN	Renal cancer	Akt downregulation; cell cycle arrest	Synergy
2	Curcumin + Doxorubicin	Osteosarcoma	JNK activation; apoptosis	Chemosensitization
3	Cucurbitacin B	Pancreatic cancer	Mitophagy; glycolysis inhibition	Multiple targets
4	Olive leaf extract	Leukemia	Apoptosis; signaling	Drug synergy
5	Grape seed oil (nano)	Solid tumor	ROS modulation	Delivery enhancement
6	Shikonin	Multiple	ROS; cell death pathways	Multiple pathways
7	Gnetin C	Prostate	Anti-inflammatory	Bioavailability issue
8	Ginseng compounds	Bone cancers	Multiple targets	Broad effects
